# Dr. Frank P. Blair

**Published:** 1904-06

**Authors:** 


Dr. Frank P. Blair, of Allegany, N. Y., a graduate of Albany Medical College, 1877, died May 21, 1904, aged 60 years.

